# Combination of Left Ventricular End-Diastolic Diameter and QRS Duration Strongly Predicts Good Response to and Prognosis of Cardiac Resynchronization Therapy

**DOI:** 10.1155/2020/1257578

**Published:** 2020-01-17

**Authors:** Zhinian Guo, Xiaoyan Liu, Xiaofeng Cheng, Chuan Liu, Ping Li, Yongming He, Rongsheng Rao, Chun Li, Yunlong Chen, Yong Zhang, Xiaoyu Luo, Jiang Wang

**Affiliations:** Institute of Cardiovascular Diseases, Xinqiao Hospital, Army Medical University (Third Military Medical University), 183 Xinqiao Street, Chongqing 400037, China

## Abstract

**Background:**

Approximately 20–40% of recipients of cardiac resynchronization therapy (CRT) do not respond to it based on the current patient selection criteria. The purpose of this study was to identify baseline parameters that can predict CRT response and to evaluate the effect of those predictive parameters on long-term prognosis.

**Methods:**

This was a retrospective, nonrandomized, noncontrolled cohort study. Patients who received CRT in our centre were divided into responders and nonresponders by the definition of CRT response (an increase in left ventricular ejection fraction (LVEF) of ≥5% and improvement of ≥1 New York Heart Association (NYHA) class from baseline to the 6-month follow-up).

**Results:**

Of the 101 patients, 68 were responders and 33 were nonresponders. Left ventricular end-diastolic diameter (LVEDD; OR: 0.88, 95% CI: 0.81–0.95, *P*=0.001) and QRS duration (OR: 1.07, 95% CI: 1.04–1.10, *P* < 0.001) were independent predictors of CRT response. The combination of LVEDD and QRS duration was more valuable for predicting CRT response (AUC 0.836; 95% CI: 0.76–0.91; *P* < 0.001). Moreover, the combination of LVEDD ≤ 71 mm and QRS duration ≥ 170 ms had a low incidence of all-cause mortality, HF hospitalisation, and the composite endpoint. In addition, baseline LVEDD had a positive correlation with QRS duration (*R*=0.199, *P*=0.046). Responders to CRT had better LV reverse remodeling.

**Conclusion:**

The combination of LVEDD and QRS duration provided more robust prediction of CRT response. Moreover, the combination of LVEDD ≤ 71 mm and QRS duration ≥ 170 ms was associated with a low incidence of all-cause mortality, HF hospitalisation, and the composite endpoint. Our results may be useful to provide individualized patient selection for CRT.

## 1. Introduction

Cardiac resynchronization therapy (CRT) is an effective therapy for heart failure (HF) patients with reduced left ventricular ejection fraction (LVEF) and intraventricular conduction delay. Large clinical trials have reported that CRT improves cardiac function, HF symptoms, exercise capacity, and quality of life as well as reduces HF-related hospitalizations and decreases mortality [1–4]. Unfortunately, the degree of response to CRT is not the same for all patients. Approximately 20–40% of patients do not show substantial benefit from CRT with the range depending on the response definition used for “nonresponders” [5]. Therefore, identifying reliable predictors of response prior to CRT implantation using noninvasive tools remains a major challenge faced by researchers. The purpose of the present study was to identify baseline parameters that can predict CRT response at the 6-month follow-up and to evaluate the effect of those predictive parameters on all-cause mortality or HF-related hospitalization.

## 2. Materials and Methods

### 2.1. Patient Selection

From January 2014 to December 2018, 118 consecutive patients with congestive HF received CRT. Patients who did not have 6 months of follow-up echocardiography to determine the changes in LVEF were not included in the study (*n*=17). Hence, a total of 101 consecutive patients were included in the present retrospective, nonrandomized, noncontrolled cohort study. The inclusion criteria were advanced HF of New York Heart Association (NYHA) class II to IV, despite an optimal medical therapy ≥3 months, left ventricular ejection fraction (LVEF) ≤ 35%, and QRS duration ≥ 130 ms. The study was approved by the Clinical Research Ethics Board of Third Military Medical University (Army Medical University). All patients who were familiar with the processes and purposes of the study agreed to participate in this study and signed an informed consent.

### 2.2. Implantation of CRT

The left ventricular (LV) lead position was selected to achieve satisfactory pacing parameters with no phrenic nerve stimulation. Via the coronary sinus, the LV lead was advanced to the lateral or posterolateral vein. If there was no accessible lateral or posterolateral vein, the great cardiac vein or middle cardiac vein was considered. The right ventricular lead was implanted at the right ventricular apex, and the right atrial lead was placed at the right atrial appendage.

### 2.3. Echocardiography

All patients underwent echocardiography before CRT implantation and at the 6-month follow-up. Images were obtained using a commercially available system (Vivid 7, General Electric-Vingmed, USA). Echocardiography parameters included LVEF (calculated using modified Simpson's formula), the area of mitral regurgitation (MR; assessed semi-quantitatively), left atrial diameter (LAD), and left ventricular end-diastolic diameter (LVEDD; measured with M-mode).

### 2.4. Definition of Left Bundle Branch Block (LBBB), Right Bundle Branch Block (RBBB), and CRT Response

LBBB was diagnosed according to conventional criteria, namely a QRS duration ≥ 120 ms with a QS or rS complex in lead V1 and a monophasic R wave with no Q waves in lead V6 [6, 7].

RBBB was defifined as a QRS duration ≥120 ms with a deep terminal S wave in leads I and V6 and an rSR', rsR', or rsr' in lead V1 or V2 [6, 7]_._

The CRT response was defined as the increase of LVEF ≥ 5% and improvement of ≥1 NYHA class from baseline to the 6-month follow-up [8, 9].

### 2.5. Definition of the Composite Endpoint

The composite endpoint was defined as all-cause mortality or hospitalization for HF.

### 2.6. Statistical Analysis

Analyses were performed using SPSS version 19.0 (SPSS Inc., USA) and MedCalc version 18.6.0 (MedCalc Inc., Belgium). Continuous variables were presented as the mean ± standard deviation or median (interquartile range). Categorical variables were presented as numbers with percentages. Differences between parametric variables were evaluated by Student's *t*-test, and differences between nonparametric variables were evaluated by the Mann–Whitney *U* test. Differences between categorical variables were evaluated by Fisher's exact test or the Chi-square test. Backward stepwise multivariate logistic regression was performed using the variables with *P* < 0.10 in the univariate logistic regression. The receiver operating characteristic (ROC) curve was used to visualize the value of variables that could independently predict response in the multivariate analysis, and the optimal cut-off value was defined as the highest level (sensitivity−(1−specificity)). Associations between LVEDD and QRS duration were assessed by linear regression analysis. Kaplan–Meier curves with log-rank tests were generated to assess significant differences in the occurrence of the endpoints. A two-sided *P* < 0.05 was considered statistically significant.

## 3. Results

### 3.1. Baseline Characteristics

Of the 101 patients (mean age of 61.22 ± 9.54 years) in the present study, 68 (67.3%) were considered responders, and 33 (32.7%) were considered nonresponders. The study subjects included 70 (69.3%) men and 31 (30.7%) women. Moreover, 62 patients had LBBB, 3 patients had RBBB, and 21 patients had intraventricular conduction delay (IVCD).

As shown in Table 1, responders had significantly smaller LAD (*P*=0.009), smaller LVEDD (*P*=0.010), and longer QRS duration (*P* < 0.001) and more frequently suffered from LBBB (*P*=0.022) than nonresponders. However, there were no significant differences in age, sex, brain natriuretic peptide (BNP), NYHA class, HF duration, hypertension, chronic renal dysfunction (CRD), ischemic cardiomyopathy (ICM), LVEF, MR, and mean follow-up time between the two groups.

### 3.2. Six-Month Follow-Up

Compared with nonresponders, responders to CRT had greater changes in LVEF, LVEDD, MR, and QRS duration from baseline to the 6-month follow-up. However, no significant differences in changes in LAD were observed between responders and nonresponders (Table 2).

### 3.3. Predictors for CRT Response

The backward stepwise multivariate analysis revealed that LVEDD (OR: 0.88, 95% CI: 0.81–0.95, *P*=0.001) and QRS duration (OR: 1.07, 95% CI: 1.04–1.10, *P* < 0.001) were independent predictors of response (Table 3).

**Figure 1:**
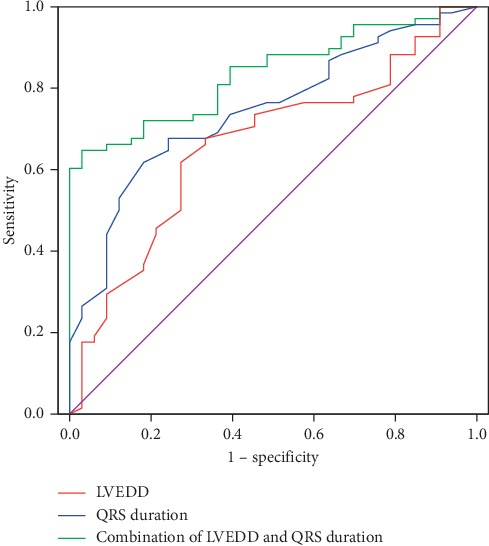
Receiver operating characteristic curve for LVEDD (red line), QRS duration (blue line), and combination of LVEDD and QRS duration (green line) in predicting response. Combination of LVEDD and QRS duration versus LVEDD alone AUC (0.836 vs. 0.662, *Z* = 3.058, *P*=0.002). Combination of LVEDD and QRS duration versus QRS duration alone AUC (0.836 vs. 0.744, *Z* = 2.309, *P*=0.021). LVEDD versus QRS duration AUC (0.662 vs. 0.744, *Z* = 1.323, *P*=0.186). LVEDD: left ventricular end-diastolic dimension; AUC: area under the curve.

ROC curve analysis showed that the area under the curve (AUC) for baseline LVEDD was 0.662 (95% CI: 0.55–0.77, *P*=0.004), with LVEDD ≤ 69 mm having 62% sensitivity, 73% specificity, 82% positive prediction value (PPV), and 48% negative prediction value (NPV). For QRS duration, the AUC was 0.744 (95% CI: 0.65–0.84, *P* < 0.001), with QRS duration ≥ 166 ms having 62% sensitivity, 82% specificity, 88% PPV, and 51% NPV. LVEDD ≤ 71 mm combined with QRS duration ≥ 170 ms had 65% sensitivity, 97% specificity, 98% PPV, and 57% NPV for predicting response (AUC 0.836; 95% CI: 0.76–0.91; *P* < 0.001). The combination of LVEDD and QRS duration was more valuable for predicting response than LVEDD (AUC, 0.836 vs. 0.662; *Z*=3.058; *P*=0.002) or QRS duration (AUC, 0.836 vs. 0.744; *Z*=2.309; *P*=0.021) alone. However, there was no difference between the value of LVEDD and QRS duration in predicting CRT response (AUC, 0.662 vs. 0.744; *Z*=1.323; *P*=0.186) (Table 4, Figure 1).

In addition, baseline LVEDD had a positive correlation with QRS duration (*R*=0.199, *P*=0.046).

### 3.4. Long-Term Prognosis

**Figure 2:**
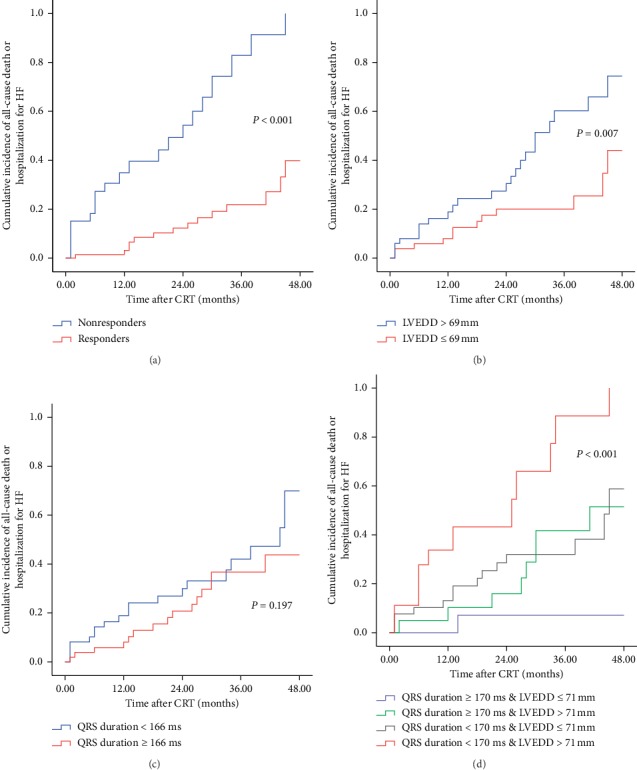
Four Kaplan–Meier curves for response category, LVEDD, QRS duration, and combination of LVEDD and QRS duration of cumulative incidence of composite endpoint at 4 years. (a) For composite endpoint, responders (red line) performed better compared to nonresponders (blue line). (b) For composite endpoint, LVEDD ≤ 69 mm group (red line) performed better compared to LVEDD > 69 mm group (blue line). (c) For composite endpoint, there was no difference between QRS duration ≤ 166 ms group and QRS duration > 166 ms group. (d) For composite endpoint, combination of QRS duration ≥ 170 ms and LVEDD ≤ 71 mm group (blue line) performed the best compared with others. HF: heart failure; LVEDD: left ventricular end-diastolic dimension.

Long-term follow-up was performed by telephone interview or clinic visit. During a mean follow-up period of 23.76 ± 14.48 months, the composite endpoint occurred in 35 patients (13 deaths and 22 hospitalizations for HF). The incidence of the composite endpoint was 40% (14 with 4 deaths and 10 hospitalizations for HF) in responders and 60% in nonrespondents (21 with 9 deaths and 12 hospitalizations). Kaplan–Meier curves showed that the cumulative incidence of the composite endpoint or all-cause mortality alone was significantly lower in responders, patients with LVEDD ≤ 69 mm, and patients with a combination of LVEDD ≤ 71 mm and QRS duration ≥ 170 ms. Moreover, the endpoint of HF hospitalisation alone was significantly less likely in responders and patients with a combination of LVEDD ≤ 71 mm and QRS duration ≥ 170 ms. However, no difference was observed between the group with QRS duration ≥ 166 ms and the group with the QRS duration < 166 ms for the cumulative incidence of the composite endpoint, all-cause mortality alone or HF hospitalisation alone (Figure 2, Supplementary Figures 1 and 2).

## 4. Discussion

In the present study, we demonstrated that LVEDD and QRS duration were independent predictors of CRT response and that the combination of LVEDD and QRS duration was more effective for response prediction. Moreover, the cumulative incidence of the composite endpoint was statistically lower in responders, LVEDD ≤ 69 mm patients and combined LVEDD ≤ 71 mm and QRS duration ≥ 170 ms patients. Moreover, baseline LVEDD had a positive correlation with QRS duration. In addition, responders to CRT had better LV reverse remodeling.

A previous study by Achilli et al. showed that a smaller LV end-systolic diameter (LVESD) is an independent predictor of a positive response to CRT, with LVESD < 60 mm having a sensitivity of 66% and a specificity of 61% [8]. Goldenberg et al. demonstrated that LV end-diastolic volume (LVEDV) is associated with CRT response [10]. In addition, Rinkuniene et al. reported that LVEDD < 75 mm is the strongest independent predictor of CRT response [11]. Díaz-Infante et al. also found that LVEDD ≥ 75 mm is an independent predictor of nonresponse to CRT [12]. In the present study, LVEDD was an independent predictor of CRT response, with LVEDD ≤ 69 mm showing 62% sensitivity and 73% specificity, which was similar to the findings from an earlier study reporting that LVEDD ≤ 67 mm is associated with CRT response after 6 months of follow-up [13]. Enlarged LV may be a marker of HF progression and impairment of contractile function [8]. Hence, patients with enlarged LVEDD were less likely to respond to CRT [12]. Moreover, Carluccio et al. found that baseline LV end-systolic volume index is a powerful predictor of events (cardiac death and hospital admission for HF) during long-term (40 ± 23 months) clinical follow-up [14]. A previous study by Adelstein et al. reported that patients with LVEDD < 3.36 cm/m height have minimal risk of appropriate shocks after a CRT-D implant [15]. In our study, patients with LVEDD ≤ 69 mm prior to the CRT implant were associated with a lower risk of the composite endpoint during a mean follow-up period of 23.76 ± 14.48 months, which confirmed previous results reporting that larger LV dimensions result in poorer prognoses [14–16].

Wider baseline QRS and a narrowing of the QRS width after CRT implantation are independent predictors of clinical positive response [17]. Another previous study reported that QRS ≥ 150 ms is associated with CRT response [10]. The PROSPECT-ECG substudy by Hsing et al. showed that QRS width predicts clinical composite score (CCS) improvement after CRT [18]. Moreover, Linde et al. studied 1591 CRT recipients and reported that QRS duration is a predictor of CRT response and that CRT delivered better benefit to patients with the QRS duration between 160 and 180 ms [19]. In the present study, the QRS duration ≥ 166 ms was considered an independent predictor of CRT response, which supported the results above. However, Mollema et al. demonstrated that baseline QRS duration is not predictive of clinical or echocardiographic response to CRT [20]. The inconsistency may be caused by different definitions of CRT response. A meta-analysis of five randomized trials has indicated that QRS duration is a powerful predictor of CRT on morbidity and mortality [21]. However, Leong et al. showed that QRS duration is not associated with death during a median follow-up of 44 months [22]. In our study, compared with patients with a QRS duration < 166 ms, patients with a QRS duration ≥ 166 ms were not associated with the cumulative incidence of the composite endpoint.

Previous studies have described a positive association between baseline QRS duration and LV size (LV length, LV diameter, and LV mass) [23–26]. Chan et al. showed that LV size increases with prolonged QRS duration in the cardiomyopathy patients [24]. Zweerink et al. reported that the normalization of QRS duration to LV dimension (i.e., QRS duration divided by LV dimension) is associated with CRT response [23]. In line with the previous reports, baseline LVEDD had a positive correlation with QRS duration (*R*=0.199, *P*=0.046) in the present study. Rickard et al. reported that there is a weak association between QRS duration and LVEDD (*R*=0.106, *P* < 0.001) [27]. However, they argued that LVEDD do not modify the effect of QRS duration, by analyzing LVEDD*∗*QRS duration (an interaction term created by Rickard et al.) in the multivariate Cox proportional hazards model [27]. Whether it is scientific and reasonable to analyze LVEDD*∗*QRS duration in the multivariate Cox proportional hazards model is warranted to confirm in future studies.

The QRS duration is typically determined by myocardial conduction velocity and conduction path length [23]. LV dilatation may result in increased conduction path length in HF patients. Hence, it is possible that the increase of LV size contributes to the prolongation of the QRS duration [25]. Increased QRS duration was found to be associated with improved CRT response. Nevertheless, progressive LV dilatation limited CRT response. Perhaps, it could be explained by the hypothesis that myocardial conduction velocity rather than conduction path length determines CRT response [23].

In the present study, the combination of LVEDD and QRS duration provided more robust prediction of CRT response than LVEDD or QRS duration alone. Moreover, the combination of LVEDD ≤ 71 mm and QRS duration ≥ 170 ms was associated with a low incidence of all-cause mortality, HF hospitalisation, or the composite endpoint. Our results may be useful to provide individualized patient selection for CRT. Further investigations are warranted to confirm these results in the future.

Prior studies have reported that LBBB, nonischemic cardiomyopathy (NICM), and female sex seemed to predict CRT response [10, 17, 18, 28, 29]. However, Linde et al. and our study found that they are not associated with CRT response [19, 30]. Moreover, female patients would have NICM and LBBB more often than male patients [31]. This inconsistency may be due to a great interobserver and intraobserver variability in these parameters.

## 5. Limitations

This study has several limitations. First, the sample size was relatively small. Thus, our results may need to be confirmed by large multicentre prospective studies in the future. Second, our study was a retrospective study, which has inherent potential limitation and may be subject to bias. Third, CRT response was defined at the 6-month follow-up, but complete LVEF recovery after CRT had been reported even after 2 years. Thus, 6-month follow-up may not allow assessment of the time course of LVEF recovery. Finally, patients who were lost at 6-month follow-up were not included in the study, which may result in selection bias.

## 6. Conclusions

The combination of LVEDD and QRS duration provided more robust prediction of CRT response. Moreover, patients with a combination of LVEDD ≤ 71 mm and QRS duration ≥ 170 ms had a low incidence of the composite endpoint. Hence, our results may be useful for identifying patients most likely to benefit from CRT.

## Figures and Tables

**Table 1:** Baseline characteristics.

Characteristics	Total population (*n*=101)	Responders (*n*=68)	Nonresponders (*n*=33)	*P* value
Age (years)	61.22 ± 9.54	61.65 ± 10.39	60.33 ± 7.55	0.473
Sex (female)	31 (30.7%)	19 (27.9%)	12 (36.4%)	0.389
BNP (ng/L)	401 (213–1185)	321 (181.3–1129)	685 (325.5–1230)	0.537
NYHA class				
II	21 (20.8%)	15 (22.1%)	6 (18.2%)	0.652
III	60 (59.4%)	40 (58.8%)	20 (60.6%)	0.864
IV	20 (19.8%)	13 (19.1%)	7 (21.2%)	0.804
HF duration (months)	48 (12–72)	30 (12–72)	60 (30–96)	0.192
Hypertension, *n* (%)	21 (20.8%)	15 (22.1%)	6 (18.2%)	0.652
CRD, *n* (%)	18 (17.8%)	11 (16.2%)	7 (21.2%)	0.535
ICM, *n* (%)	17 (16.8%)	12 (17.6%)	5 (15.2%)	0.756
LVEF (%)	29.40 ± 4.42	29.56 ± 4.10	29.08 ± 5.06	0.609
LAD (mm)	44.39 ± 5.48	43.41 ± 5.20	46.43 ± 5.58	**0.009**
LVEDD (mm)	70.06 ± 7.84	68.68 ± 7.38	72.91 ± 8.12	**0.010**
MR (cm^2^)	7.2 (4.5–10.4)	7.2 (4.5–10.4)	7.5 (4.4–10.4)	0.278
QRS duration (ms)	165.30 ± 21.33	171.20 ± 21.56	153.30 ± 15.07	**< 0.001**
LBBB, *n* (%)	62 (61.4%)	47 (69.1%)	15 (45.5%)	**0.022**
Mean follow-up time (months)	23.76 ± 14.48	23.60 ± 14.83	24.09 ± 13.95	0.875

Values are mean ± SD, median (range) or *n* (%). BNP: brain natriuretic peptide; NYHA: New York Heart Association; HF: heart failure; CRD: chronic renal dysfunction; ICM: ischemic cardiomyopathy; LVEF: left ventricular ejection fraction; LAD: left atrial dimension; LVEDD: left ventricular end-diastolic dimension; MR: mitral regurgitation; LBBB: left bundle branch block.

**Table 2:** Changes in echocardiography and electrocardiogram from baseline to 6 months in two groups.

Variables	Responders (*n*=68)	Nonresponders (*n*=33)	*P* value
Change in LVEF	12 (8–20)	2 [(−4)–4]	**< 0.001**
Change in LAD	−3.2 [(−6)–0]	−3.5 [(−7)–1.5]	0.649
Change in LVEDD	−6 [(−15)–(−2)]	0 [(−3.5)–4.5]	**< 0.001**
Change in MR	−4.5 [(−7.2)–(−1.6)]	−2.1 [(−5.1)–2.2]	**0.008**
Change in QRS duration	−34.13 ± 25.69	−19.26 ± 24.81	**0.009**

LVEF: left ventricular ejection fraction; LAD: left atrial dimension; LVEDD: left ventricular end-diastolic dimension; MR: mitral regurgitation.

**Table 3:** Univariate and backward stepwise multivariate logistic regression analyses with regard to predictors of response.

	Univariate analysis		Multivariate analysis	
Baseline characteristics	OR (95% CI)	*P* value	OR (95% CI)	*P* value
Age	1.02 (0.97–1.06)	0.515		
Sex	1.47 (0.61–3.57)	0.391		
BNP	1.00 (1.00–1.00)	0.533		
NYHA class	0.86 (0.45–1.66)	0.659		
HF duration	1.00 (0.99–1.00)	0.196		
Hypertension	1.27 (0.44–3.25)	0.653		
CRD	0.72 (0.25–2.06)	0.536		
ICM	1.20 (0.39–3.74)	0.753		
LVEF	1.03 (0.93–1.13)	0.605		
LAD	0.90 (0.83–0.98)	**0.012**	0.94 (0.85–1.04)	0.237
LVEDD	0.93 (0.88–0.99)	**0.014**	0.88 (0.81–0.95)	**0.001**
MR	0.96 (0.89–1.04)	0.293		
QRS duration	1.05 (1.03–1.08)	<**0.001**	1.07 (1.04–1.10)	**<0.001**
LBBB	2.69 (1.14–6.33)	**0.024**	1.71 (0.60–4.88)	0.315

OR: odds ratio; 95% CI: 95% confidence interval. BNP: brain natriuretic peptide; NYHA: New York Heart Association; HF: heart failure; CRD: chronic renal dysfunction; ICM: ischemic cardiomyopathy; LVEF: left ventricular ejection fraction; LAD: left atrial dimension; LVEDD: left ventricular end-diastolic dimension; MR: mitral regurgitation; LBBB: left bundle branch block.

**Table 4:** AUC and cut-off value for LVEDD, QRS duration, and combination of LVEDD and QRS duration.

Variables	AUC	95% CI	*P* for AUC	Cut-off value	Sensitivity (%)	Specificity (%)	PPV (%)	NPV (%)
LVEDD (mm)	0.662	0.55–0.77	**0.004**	≤69	62	73	82	48
QRS duration (ms)	0.744	0.65–0.84	<**0.001**	≥166	62	82	88	51
Combination of LVEDD and QRS duration (mm, ms)	0.836	0.76–0.91	<**0.001**	LVEDD ≤ 71 and ≥170	65	97	98	57

LVEDD: left ventricular end-diastolic dimension; AUC: area under the curve; 95% CI: 95% confidence interval; PPV: positive prediction value; NPV: negative prediction value.

## Data Availability

The data used to support the findings of this study are available from the corresponding author upon request.

## Supplementary Materials

Supplementary Materials Supplemental Figure 1. Four Kaplan–Meier curves for response category, LVEDD, QRS duration, and combination of LVEDD and QRS duration of cumulative incidence of all-cause death at 4 years. (A) For cumulative incidence of all-cause death, responders (red line) performed better compared with nonresponders (blue line). (B) For cumulative incidence of all-cause death, the LVEDD ≤ 69 mm group (red line) performed better compared with the LVEDD > 69 mm group (blue line). (C) For cumulative incidence of all-cause death, there was no difference between the QRS duration ≤ 166 ms group and QRS duration > 166 ms group. (D) For cumulative incidence of all-cause death, combination of QRS duration ≥ 170 ms and the LVEDD ≤ 71 mm group performed the best compared with others. LVEDD: left ventricular end-diastolic dimension. Supplemental Figure 2. Four Kaplan–Meier curves for response category, LVEDD, QRS duration, and combination of LVEDD and QRS duration of cumulative incidence of hospitalization for heart failure at 4 years. (A) For cumulative incidence of hospitalization for heart failure at 4 years, responders (red line) performed better compared with nonresponders (blue line). (B) For cumulative incidence of hospitalization for heart failure, there was no difference between the LVEDD ≤ 69 mm group and LVEDD > 69 mm group (red line). (C) For cumulative incidence of hospitalization for heart failure, there was no difference between the QRS duration ≤ 166 ms group and QRS duration > 166 ms group. (D) For cumulative incidence of hospitalization for heart failure, the combination of QRS duration ≥ 170 ms and LVEDD ≤ 71 mm group (blue line) performed the best compared with others. LVEDD: left ventricular end-diastolic dimension. .
