# Supplementary material for: Combination of Left Ventricular End-Diastolic Diameter and QRS Duration Strongly Predicts Good Response to and Prognosis of Cardiac Resynchronization Therapy
Source: Cardiol Res Pract. 2020 Jan 17;2020:1257578. doi: 10.1155/2020/1257578 (PMC7201746; doi:10.1155/2020/1257578)
Supplement: Supplementary Materials — Supplemental Figure 1. Four Kaplan–Meier curves for response category, LVEDD, QRS duration, and combination of LVEDD and QRS duration of cumulative incidence of all-cause death at 4 years. (A) For cumulative incidence of all-cause death, responders (red line) performed better compared with nonresponders (blue line). (B) For cumulative incidence of all-cause death, the LVEDD ≤ 69 mm group (red line) performed better compared with the LVEDD > 69 mm group (blue line). (C) For cumulative incidence of all-cause death, there was no difference between the QRS duration ≤ 166 ms group and QRS duration > 166 ms group. (D) For cumulative incidence of all-cause death, combination of QRS duration ≥ 170 ms and the LVEDD ≤ 71 mm group performed the best compared with others. LVEDD: left ventricular end-diastolic dimension. Supplemental Figure 2. Four Kaplan–Meier curves for response category, LVEDD, QRS duration, and combination of LVEDD and QRS duration of cumulative incidence of hospitalization for heart failure at 4 years. (A) For cumulative incidence of hospitalization for heart failure at 4 years, responders (red line) performed better compared with nonresponders (blue line). (B) For cumulative incidence of hospitalization for heart failure, there was no difference between the LVEDD ≤ 69 mm group and LVEDD > 69 mm group (red line). (C) For cumulative incidence of hospitalization for heart failure, there was no difference between the QRS duration ≤ 166 ms group and QRS duration > 166 ms group. (D) For cumulative incidence of hospitalization for heart failure, the combination of QRS duration ≥ 170 ms and LVEDD ≤ 71 mm group (blue line) performed the best compared with others. LVEDD: left ventricular end-diastolic dimension. . [file 1257578.f1.pdf]

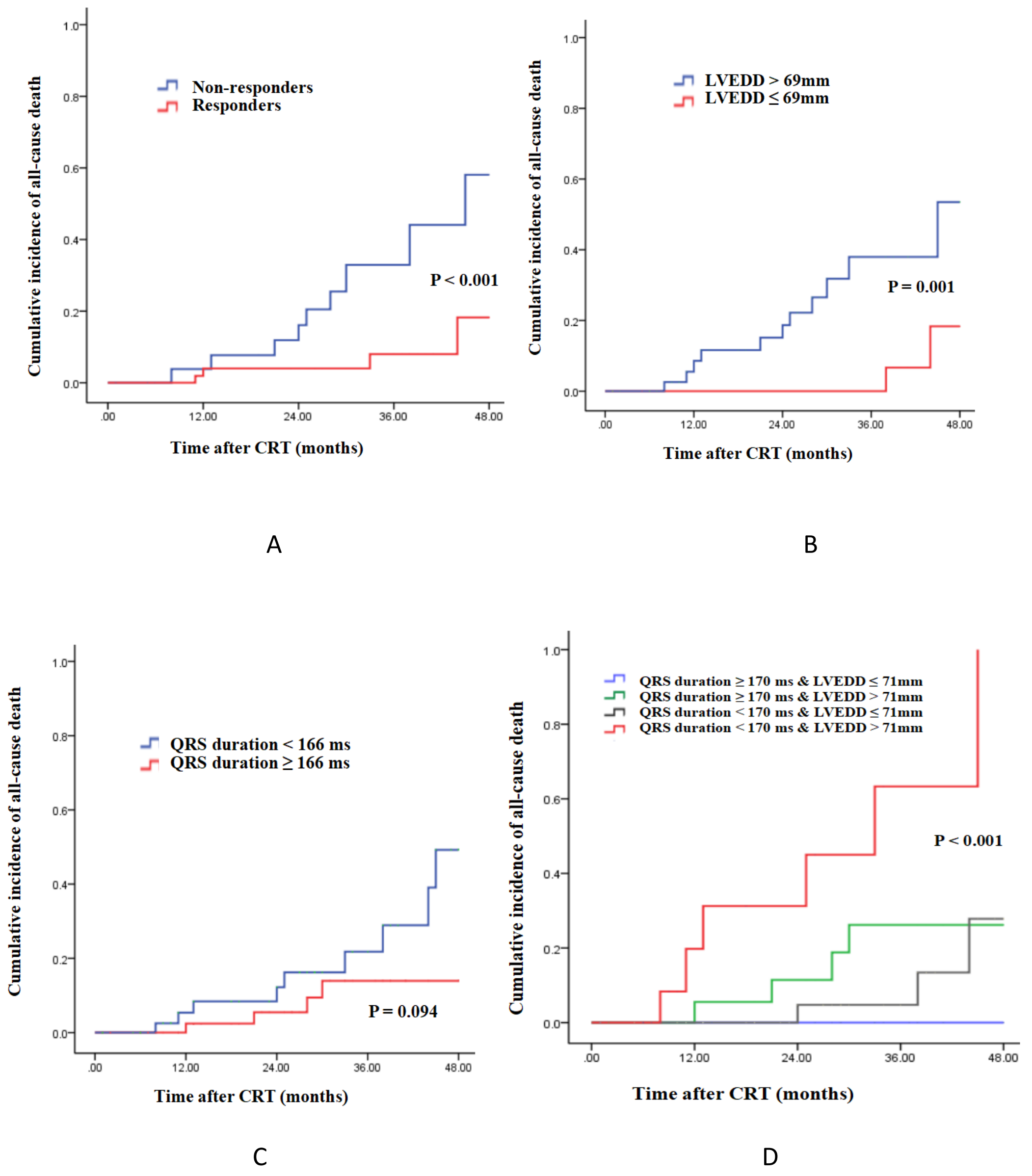


**Supplemental Figure 1.** Four Kaplan-Meier curves for response category, LVEDD, QRS duration and combination of LVEDD and QRS duration of cumulative incidence of all-cause death at 4 years. (A) For cumulative incidence of all-cause death, responders (red line) performed better compared to non-responders (blue line). (B) For cumulative incidence of all-cause death, LVEDD ≤ 69mm group (red line) performed better compared to LVEDD > 69mm group (blue line). (C) For cumulative incidence of all-cause death, there was no difference between QRS duration ≤ 166ms group and QRS duration > 166ms group. (D) For cumulative incidence of all-cause death, combination of QRS duration ≥ 170 ms and LVEDD ≤ 71mm group performed the best compared to others.

LVEDD: left ventricular end-diastolic dimension

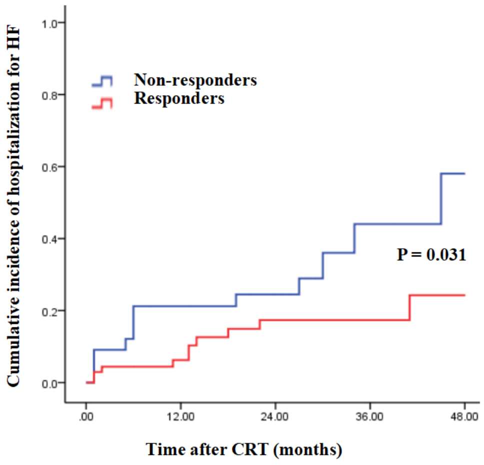


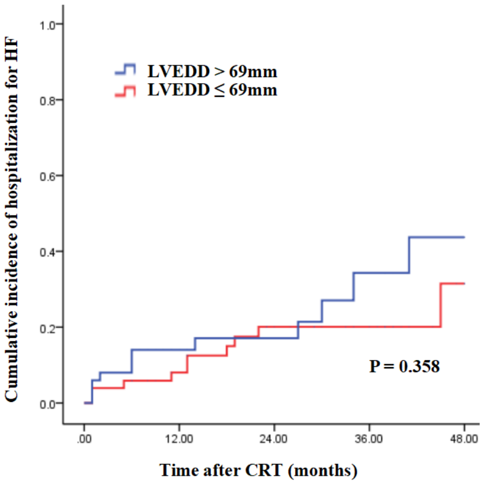


A B

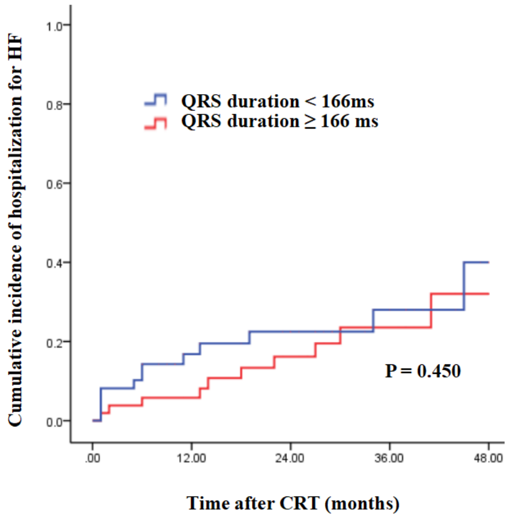


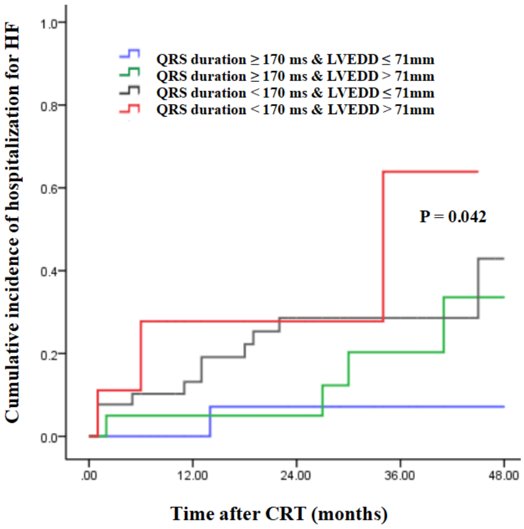


C D

**Supplemental Figure 2.** Four Kaplan-Meier curves for response category, LVEDD, QRS duration and combination of LVEDD and QRS duration of cumulative incidence of hospitalization for heart failure at 4 years. (A) For cumulative incidence of hospitalization for heart failure at 4 years, responders (red line) performed better compared to non-responders (blue line). (B) For cumulative incidence of hospitalization for heart failure, there was no difference between LVEDD ≤ 69mm group and LVEDD > 69mm group (red line). (C) For cumulative incidence of hospitalization for heart failure, there was no difference between QRS duration ≤ 166ms group and QRS duration > 166ms group. (D) For cumulative incidence of hospitalization for heart failure, combination of QRS duration ≥ 170 ms and LVEDD ≤ 71mm group (blue line) performed the best compared to others.

LVEDD: left ventricular end-diastolic dimension
